# The lethal effect of soap on *Schistosoma mansoni* cercariae in water

**DOI:** 10.1371/journal.pntd.0012372

**Published:** 2024-07-29

**Authors:** Jiaodi Zhang, Ana K. Pitol, Safari Kinung’hi, Teckla Angelo, Aidan M. Emery, Adam Cieplinski, Michael R. Templeton, Laura Braun

**Affiliations:** 1 Department of Civil and Environmental Engineering, South Kensington Campus, Imperial College London, London, United Kingdom; 2 Department of Vector Biology, Liverpool School of Tropical Medicine, Liverpool, United Kingdom; 3 National Institute for Medical Research (NIMR), Mwanza Centre, Mwanza, Tanzania; 4 School of Life Science and Bioengineering, Nelson Mandela African Institution of Science and Technology, Arusha, Tanzania; 5 Wolfson Wellcome Biomedical Laboratories, Natural History Museum, London, United Kingdom; 6 Department of Disease Control, London School of Hygiene and Tropical Medicine, London, United Kingdom; Huazhong University of Science and Technology Tongji Medical College, CHINA

## Abstract

**Background:**

Schistosomiasis is a parasitic disease which is spread through skin contact with water containing *Schistosoma* cercariae. Drug treatment has been the main control method, but it does not prevent reinfection. The use of soap can be a complementary measure to reduce transmission. Therefore, this study investigates the quantitative effect of different soaps on the mortality of *Schistosoma mansoni* cercariae.

**Methodology:**

Four soaps including two powder soaps (Kleesoft and Omo) and two bar soaps (B29 and Rungu) which are used in a schistosomiasis-endemic Tanzanian village were studied. *S*. *mansoni* cercariae were exposed to powder soaps of 0 (control), 10, 50, 75, 100 and 1000 mg/L and to bar soaps of 0 (control), 100, 500 and 1000 mg/L. The highest concentration of 1000 mg/L was selected based on the laboratory-estimated average soap concentration during handwashing. Cercariae were observed under a microscope after 0, 5, 15, 30, 45 and 60 minutes of exposure to determine their survival.

**Conclusions:**

All four soaps can kill *S*. *mansoni* cercariae and this lethal effect was related to soap concentration and exposure time. At the highest concentration of 1000 mg/L, all cercariae were dead at 5 minutes post-exposure with two powder soaps and Rungu, while 100% cercarial death was achieved between 5 minutes to 15 minutes for B29. Almost all cercariae survived after being exposed to 10 mg/L powder soaps and 100 mg/L bar soaps for 60 minutes. Powder soaps were more lethal than bar soaps. Considering the widely varying concentrations of soap during real-world hygiene activities and the necessity for a very high soap concentration to eliminate all cercariae in a short 5-minute exposure, providing the efficacy of soap in preventing schistosomiasis becomes challenging. Future studies should investigate whether soap can influence alternative mechanisms such as making cercariae unable to penetrate the skin, thereby providing protection.

## Author summary

Schistosomiasis is a major public health problem in low-resource countries where there is insufficient access to safe water, sanitation and hygiene (WASH). Preventive chemotherapy with praziquantel has been the main control method, but it cannot protect against reinfection. With the increasing awareness of the importance of a multi-faceted control strategy, WASH has been recommended as one of the core strategic interventions for schistosomiasis control. The use of soap, one hygiene practice, might play a role in prevention by reducing the penetration of cercariae into human skin during water contact, thereby lowering the infection risk at transmission sites. However, to the best of our knowledge, there are no detailed guidelines on how to use soap to prevent schistosomiasis in endemic areas. Therefore, this study aimed to explore the efficacy of four commercial Tanzanian soaps against *Schistosoma mansoni* cercariae to understand soap lethality. The results provide scientific evidence of soap protection against schistosome cercariae and suggest that future studies should investigate other potential mechanisms by which soap may provide protection against cercariae (e.g. damaging them sufficiently to hinder penetration into the skin or protecting skin against cercarial penetration).

## Introduction

Schistosomiasis is a neglected tropical disease (NTD) caused by parasitic worms of the genus *Schistosoma*. This disease is endemic in 78 countries, primarily in regions lacking safe access to water, sanitation and hygiene (WASH) services [1]. According to the estimation by the Global Burden of Disease 2019, schistosomiasis caused 1.6 million disability-adjusted life years (DALYs), ranking the third largest contributor to the DALYs caused by NTDs after dengue and intestinal nematode infections [2].

*Schistosoma* infection occurs via skin contact with water contaminated with schistosome cercariae, the larval stage of schistosomes. Cercariae emerge from the freshwater snails which act as intermediate hosts. After locating the human host, cercariae penetrate human skin, develop into adult worms and mate to produce eggs which are released from infected individuals into water via feces or urine, depending on *Schistosoma* species. Eggs hatch into free-living miracidia which infect the intermediate hosts (snails), and the transmission cycle of the schistosome parasite continues.

The most widely used intervention to interrupt transmission of schistosomiasis is mass drug administration with praziquantel [3]; however, reinfection is likely if skin contact with water containing cercariae continues. Instead of one single intervention, a more comprehensive approach emphasising the importance of WASH interventions has been promoted to achieve schistosomiasis elimination as a public health problem [3–5]. Some studies have explored the possibility of improving water infrastructure using effective water treatment methods, such as chlorine and UV disinfection, to provide safe and cercaria-free water at household and community levels [6–10]. Hygiene, a component of WASH, has also been recommended by the World Health Organization (WHO) as one of the core strategic interventions for schistosomiasis control, and individual hygiene education (e.g. safe sanitation access, personal hygiene) is proposed [1]. However, the use of soap during water contact, one important part of good hygiene, is not specifically mentioned.

Soap is a common hygiene product that people may use during daily washing activities, such as laundry, bathing, dishwashing, and handwashing. These water-contact activities are associated with the risk of schistosomiasis infection and have been shown to vary with age and gender [11–13]. For laundry, generally only hands and feet are exposed to water, while the whole body is exposed to water during bathing. Soap comes in three main physical forms: powder, bar, or liquid soap. Soap is chemically defined as the alkali salt of fatty acids and made by the saponification reaction of a base and with animal or vegetable fats [14,15]. As the performance of soap can be hindered in saltwater or hard water (i.e. containing high concentration of calcium and magnesium ions), and insoluble salts may remain on the washed surface (e.g. clothes or skin), synthetic surfactants which avoid these drawbacks have been introduced into soap manufacture [14,16]. The commercial soap and detergent now are a mixture of surfactants, builders, blenches, enzymes, perfumes, among other ingredients [14,16].

If effective against schistosome cercariae, using soap could be a control measure against schistosomiasis since transmission to humans occurs when cercariae successfully penetrate human skin during water contact. Hence, it is worth understanding the protective effect that soap provides under different conditions.

The use of soap can help prevent many infectious diseases [17–20], but there is limited knowledge about its effect against schistosome cercariae. A systematic review has demonstrated that soap has the potential to reduce the *Schistosoma* infection risk, with one protective mechanism being that soap kills schistosome cercariae [21]. This lethal effect was associated with both the concentration of soap and the exposure time [21–27]. However, many studies recorded only the time required to reach 100% mortality [23–25], resulting in prolonged exposure durations of up to 12 hours, which may not reflect real life scenarios. Therefore, it is important to test shorter exposure times based on the durations of different water contact activities involving soap (e.g. laundry, bathing) in endemic areas to understand soap’s protective effects. Additionally, two studies reported low number of cercariae (e.g. only 10 cercariae per sample) [26,28], making the results less informative.

To improve the understanding of the effect of soap on cercarial mortality, this research study was carried out to investigate the lethal effect of different soaps on *S*. *mansoni* cercariae under laboratory conditions. This study used *S*. *mansoni* cercariae, which are one of the main *Schistosoma* species infecting humans [29,30]. Experiments involving soap were conducted using powder and bar soaps used in a schistosomiasis-endemic village of Tanzania to quantitatively determine the cercaria death based on the motility and morphology at different soap concentrations and different exposure times from a few minutes up to 1 hour.

## Methods

### Ethics statement

In Tanzania, this study was approved by the Tanzania Medical Research Coordination Committee (MRCC) of the National Institute for Medical Research (NIMR), and the ethics approval certificate number is NIMR/HQ/R.8a/Vol. IX/2610.

### Egg source

Eggs of *Schistosoma mansoni* (NMRI) were provided by Schistosomiasis Resource Center (SRC) of the National Institute of Allergy and Infectious Diseases (NIAID), Rockville, USA, and collected from livers of Swiss Webster female mice (seven weeks post infection). SRC has full Association for Assessment and Accreditation of Laboratory Animal Care (AAALAC) accreditation (Site 000779), operating under the National Institutes of Health’s Office of Laboratory Animal Welfare (OLAW) with the assurance ID D16-00046. Eggs were shipped to the Natural History Museum, London, UK in RPMI / 1X Pen / Strep / 2.0% FBS.

### Snail infection

Snail infections were performed at the Natural History Museum (London, UK) with *S*. *mansoni*. Eggs were hatched into miracidia in artificial spring water [31] which was also used for snail maintenance at the Natural History Museum. *Biomphalaria glabrata* snails were infected with 10 miracidia individually in plastic cups containing 25 mL of artificial spring water and left overnight. Snails were transferred to the Roger Perry Laboratory at Imperial College London (London, UK) and kept in Volvic bottled water at 27°C. Cercarial production started 25–30 days post-infection. Snails were kept in the dark for at least 24 hours before all experiments to achieve a good shedding of cercariae.

### Cercarial preparation and enumeration

The preparation and enumeration of cercariae were performed using a similar procedure of previous research [7,8,32]. Three to nine infected snails were randomly selected and rinsed in bottled water. Then, these snails were transferred to a beaker containing 3–9 mL of bottled water (pH 7, 27°C). The beaker containing snails was placed under bright light for 1–1.5 hour to obtain cercariae. The snails were removed and the cercaria solution was filtered through a 200 μm polyester mesh to remove snail feces. To quantify the number of cercariae in the water, three 100 μL aliquots were taken by pipette and 20 μL of Lugol solution (Sigma Aldrich) was added to kill and stain cercariae for counting. After calculating the average concentration based on the three aliquots, the volume of cercarial solution required to achieve 100 cercariae per sample was determined. Fresh cercariae were prepared daily and all experiments were completed within a maximum of six hours of the cercariae which have been shed from snails.

### Soap use information

In 2021, a survey was carried out with ten village members (five community members, one village chairman, two subvillage leaders and two shop vendors selling soap) about the soaps used and the usage purposes for each soap type in Mwankalima village in Tanzania. Mwakalima village was chosen since this village is schistosomiasis-endemic and NIMR is conducting ongoing research. Our village survey demonstrated that powder soap and bar soap are used by local people. Therefore, two powder soaps (Kleesoft and Omo) and two bar soaps (B29 and Rungu) which are used by local people living in this village were obtained. The information of these four soaps is summarised in Table 1. The bar soap Rungu is an anti-bacterial soap which has the active ingredient, irgasan DP 300. This ingredient is also named as triclosan which is a common anti-bacterial chemical used in hygiene products [33].

**Table 1:** The information of four soaps tested in this study.

Soap name	Soap type	Soap components	Main usage purposes
Kleesoft	Powder	Surfactants, sodium tripolyphosphate, sodium sulfate, sodium carbonate, sodium silicate, optical brighteners (CBS), proteolytic enzymes, perfumes	Laundry, kitchen dishwashing
Omo		Surfactants, builders, silicates, anti-redeposition agents, enzymes, perfume, optical brighteners	
B29	Bar	No ingredient information available	Laundry, bathing, kitchen dishwashing
Rungu		Active ingredient: irgasan DP300	

### Soap exposure procedure

An experiment was conducted to understand the average soap concentration when washing hands, and detailed methods are presented in S1 File. It was estimated that the average soap concentration during handwashing was 1075±223 mg/L, which was determined as the highest concentration tested in this study. The lowest concentrations were selected as 10 mg/L for powder soap and 100 mg/L for bar soap since the preliminary result showed that > 99% of cercariae survived after exposed to these concentrations for 60 minutes. The following concentrations of powder and bar soaps were tested: (1) for powder soap: 0 (control), 10, 50, 75, 100 and 1000 mg/L; (2) for bar soap: 0 (control), 100, 500, 1000 mg/L. Different concentrations were tested for two soap types to show the varied soap lethality.

For every experiment, a fresh soap stock solution of 2000 mg/L was prepared by dissolving 0.1 g soap in 50 mL Volvic bottled water using a hotplate stirrer and the solution was allowed to cool down. For the sample preparation, a similar procedure in a previous study on chlorination of cercariae was used [8]. Approximately 100 cercariae suspended in bottled water were added to a 10 mL beaker with the desired concentration of soap to make up a total volume of 2 mL. All samples were prepared using Volvic bottled water which is a brand of mineral water and has been used as the water source in previous cercaria-related studies [7,8]. This bottled water contains mineral components such as calcium, magnesium, sodium, sulfates and chlorides which commonly exist in real water bodies [34–38] and which are also included in the recipe of artificial spring water suitable for freshwater snails [31]. The concentrations of calcium and magnesium are 12 and 8 mg/L respectively [38], hence the water hardness (CaCO_3_) in Volvic was 62.9 mg/L, within the range of water hardness that has been reported in African rivers and lakes [36,37].

### Enumeration of cercariae post-exposure

Based on the village survey conducted in Tanzania, laundry has the maximum water contact duration of up to 1 hour; therefore, 60 minutes was selected as the longest exposure time in this study. For all concentrations of two powder soaps, 100 mg/L bar soaps and control samples (0 mg/L), a beaker containing the desired soap concentration was examined after 0, 5, 15, 30, 45 and 60 minutes of exposure under a stereomicroscope to observe cercariae. For 500 mg/L and 1000 mg/L bar soaps, different beakers containing the desired soap concentrations were prepared for each exposure time because the cercaria-soap solution was required to be diluted for easier observation of dead cercariae (details of the dilution are described later in the section). For 1000 mg/L powder and bar soaps, exposure times longer than the duration when all cercariae died were not tested.

Both motility and morphology of cercariae were studied when determining whether the cercariae were alive or dead. Similar to the criteria developed by a previous study [7], a cercaria was determined as dead if it showed all four characteristics: 1) a cessation of motility, 2) everted suckers, 3) a fully relaxed tail, 4) a slightly sharp edge of the head-tail-junction on the head side. Photos showing the characteristics of dead and living cercariae can be found in S2 File. To avoid double counting, only heads were counted if heads and tails were separated.

As discussed in a previous study, cercariae fall to the bottom when they lose motility; therefore, only cercariae on the bottom were studied to obtain the number of dead cercariae [7]. For experiments on all concentrations of two powder soaps, 100 mg/L bar soaps and control samples (0 mg/L), the same method was used. Only the cercariae on the bottom of the beaker were examined and the ones which met the criteria described above were considered as dead and counted. For experiments on bar soaps of 500 mg/L and 1000 mg/L, the cercaria-soap solution was diluted for easier observation of dead cercariae due to the solution cloudiness and floc formation. For example, samples of 500 mg/L were diluted to 100 mg/L by adding Volvic water into the 10 mL beaker to stop the reaction since almost all cercariae survived at 60 minutes post-exposure to this concentration. Then, the diluted solution was transferred to a 50 mL beaker. Beakers were rinsed with Volvic water to ensure that all cercariae were collected. As some cercariae were observed to be dead on the surface of the diluted solution in the preliminary test, dead cercariae in all layers of both beakers were studied for the diluted samples. For samples of bar soaps at 1000 mg/L, whether there were any cercariae alive was determined instead of counting dead cercariae because of the difficulty of counting cercariae in cloudy soap solutions.

At the end of exposure, Lugol solution was added to fix and stain cercariae, and the solution were examined to obtain the total number of cercariae.

All experimental conditions and replicates were summarised in Table 2. Different experimental conditions were tested in a randomised order. For each experimental condition, four independent replicates were conducted, which mean that these replicates were tested on different days using freshly prepared soap solutions every time.

**Table 2:** Summary of experimental conditions, the number of replicates per experimental condition and the number of samples.

Soap type	Soap name	Soap concentration (mg/L)	Exposure time (minutes)	# experimental conditions	# replicates per condition	# samples
Powder	Kleesoft	10, 50, 75, 100	0, 5, 15, 30, 45, 60	4*6 = 24	4	96
		1000	0, 5	1*2 = 2		8
	Omo	10, 50, 75, 100	0, 5, 15, 30, 45, 60	4*6 = 24	4	96
		1000	0, 5	1*2 = 2		8
Bar	B29	100, 500	0, 5, 15, 30, 45, 60	2*6 = 12	4	48
		1000	0, 5, 15	1*3 = 3		12
	Rungu	100, 500	0, 5, 15, 30, 45, 60	2*6 = 12	4	48
		1000	0, 5	1*2 = 2		8
Control		0	0, 5, 15, 30, 45, 60	1*6 = 6	24	144

### Statistical analysis

The number of cercariae per sample ranged from 76 to 131. The percentage of dead cercariae was calculated using the following equation to account for natural cercaria death based on control samples. Although cercariae rarely died in control samples, with cercaria mortalities in control samples below 2% at all time points evaluated (0 to 60 min), this correction was still applied to the observed percentage of cercariae death in soap samples.:


$$Percentage\;of\;dead\;cercariae\;(\%)=\left(\frac{Number\;of\;dead\;cercariae\;in\;the\;soap\;sample}{Total\;number\;of\;cercariae\;in\;the\;soap\;sample}-\frac{Number\;of\;dead\;cercariae\;in\;the\;control\;sample\;}{Total\;number\;of\;cercariae\;in\;the\;control\;sample}\right)\times 100$$

The calculated percentage of dead cercariae can be below 0 when the same number of cercariae died in both the soap and control samples while the total number of cercariae in the soap sample was slightly higher than that of the control sample, which results in a higher percentage of dead cercariae in the control sample. In this case, the mortality rate was adjusted to 0.

Since all cercariae were observed to be dead when exposed to 100 mg/L powder soap for 60 minutes, the number of dead cercariae counted at 60 minutes post-exposure was used as the total number of cercariae in the sample to obtain the percentage of dead cercariae at each exposure time.

Statistical analysis was conducted using SPSS software (Version 29, IBM). The percentages of dead cercariae under different experimental conditions were compared using the Mann-Whitney *U* test. This non-parametric test was used because of the small sample size. The results were considered significant at *p*-value < 0.05.

When comparing the results with previous studies, 1 ppm is converted to 1 mg/L assuming that the liquid density is 1 g/mL.

## Results

### Powder soap

**Fig 1:**
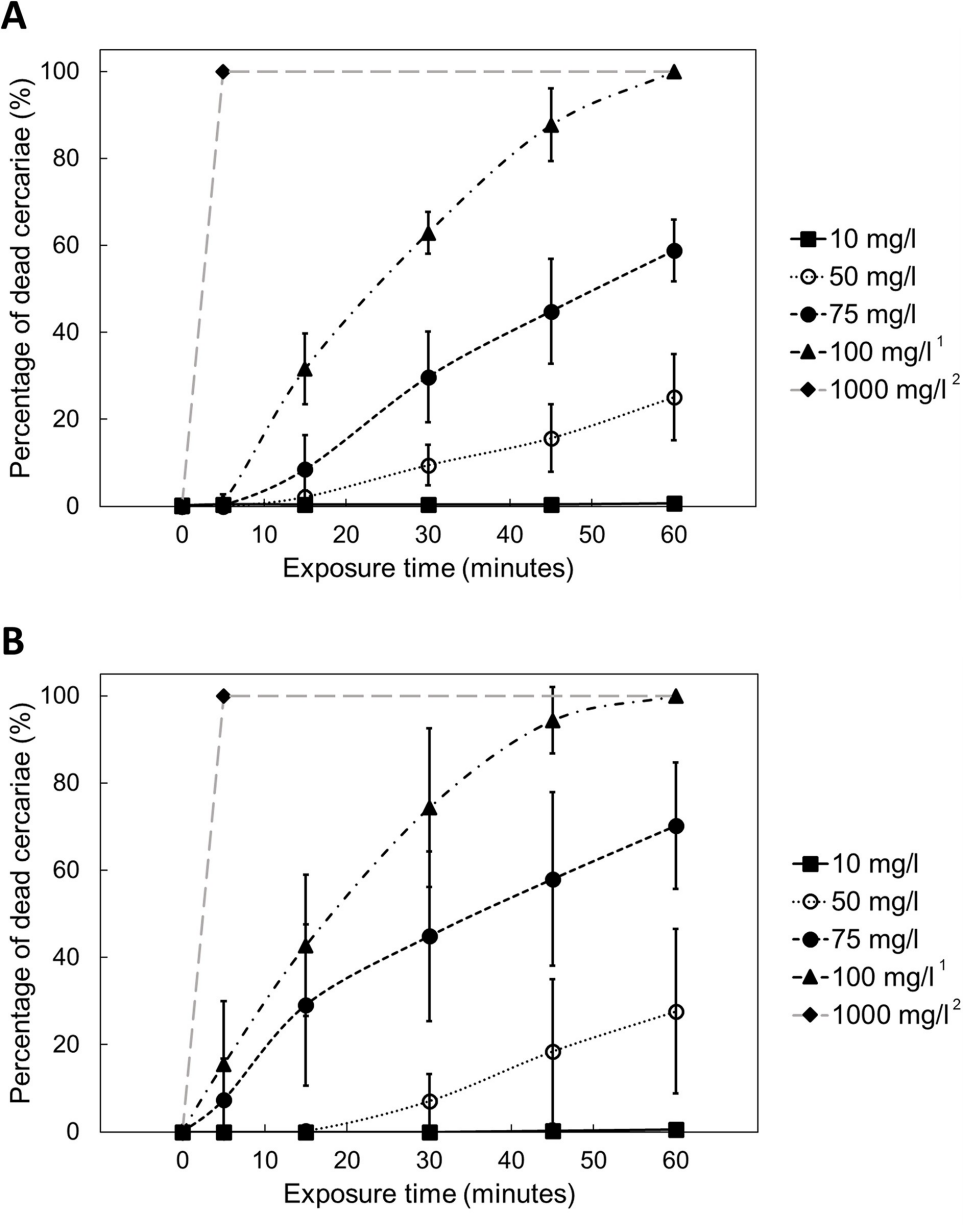
**The percentage of dead *S*. *mansoni* cercariae at different concentrations and exposure times of two powder soaps: (A) Kleesoft and (B) Omo.** Each data point represents the average of four replicates with the error bar indicating ±1 standard deviation. ^1^ indicates that all cercariae died at 60 minutes post-exposure and the number of dead cercariae counted at 60 minutes was used as the total number of the sample to obtain the mortality percentage at each exposure time.^2^ The long dashed grey line was used to connect data points of 0 and 5 minutes at 1000 mg/L and was extended to 60 minutes, as all cercariae were dead at 5 minutes of exposure.

The effect of two powder soaps was investigated: Kleesoft and Omo. The average percentage of dead cercariae at each experimental condition is presented in Fig 1. For these two soaps at 1000 mg/L, cercariae were observed to immediately move more slowly once in contact with soap and sink to the bottom of the beaker within 1 minute, though they were not completely dead as they did not meet all four criteria for death. All cercariae were found dead at 5 minutes post-exposure at this highest concentration. At 10 mg/L of both powder soaps, all cercariae were alive and almost all of them swam actively near the solution surface rather than laying on the bottom of the beaker.

As shown in Fig 1, at concentrations of 50, 75 and 100 mg/L, the percentage of dead cercariae increased with exposure time at the same concentration. The mortality curves at 50 mg/L for both powder soaps illustrate a slower increase before 15 minutes in comparison with the noticeable rise after this exposure time, suggesting the importance of exposure time at the low concentration and the possible temporary resistance against low concentration of soap. This slower increase in cercaria mortality is also shown between 45- and 60-minute post-exposure to 100 mg/L both powder soaps, which might be because all cercariae died just before 60 minutes. The high standard deviation of the data shown in Fig 1 indicates a high variability in the efficacy of soap against cercariae. These data were derived from four independent replicates and each replicate was conducted with a different batch of cercariae.

For both soaps at 100 mg/L, all cercariae died at 60 minutes post-exposure. At lower concentration of 50 mg/L, the percentage of dead cercariae gradually increased with the increase in exposure time, and finally reached around 26% for two soaps. Omo appears to more lethal at 75 mg/L than Kleesoft, indicated by a higher mortality rate of 70% at 60 minutes post-exposure compared to 59% for Kleesoft.

### Bar soap

**Fig 2:**
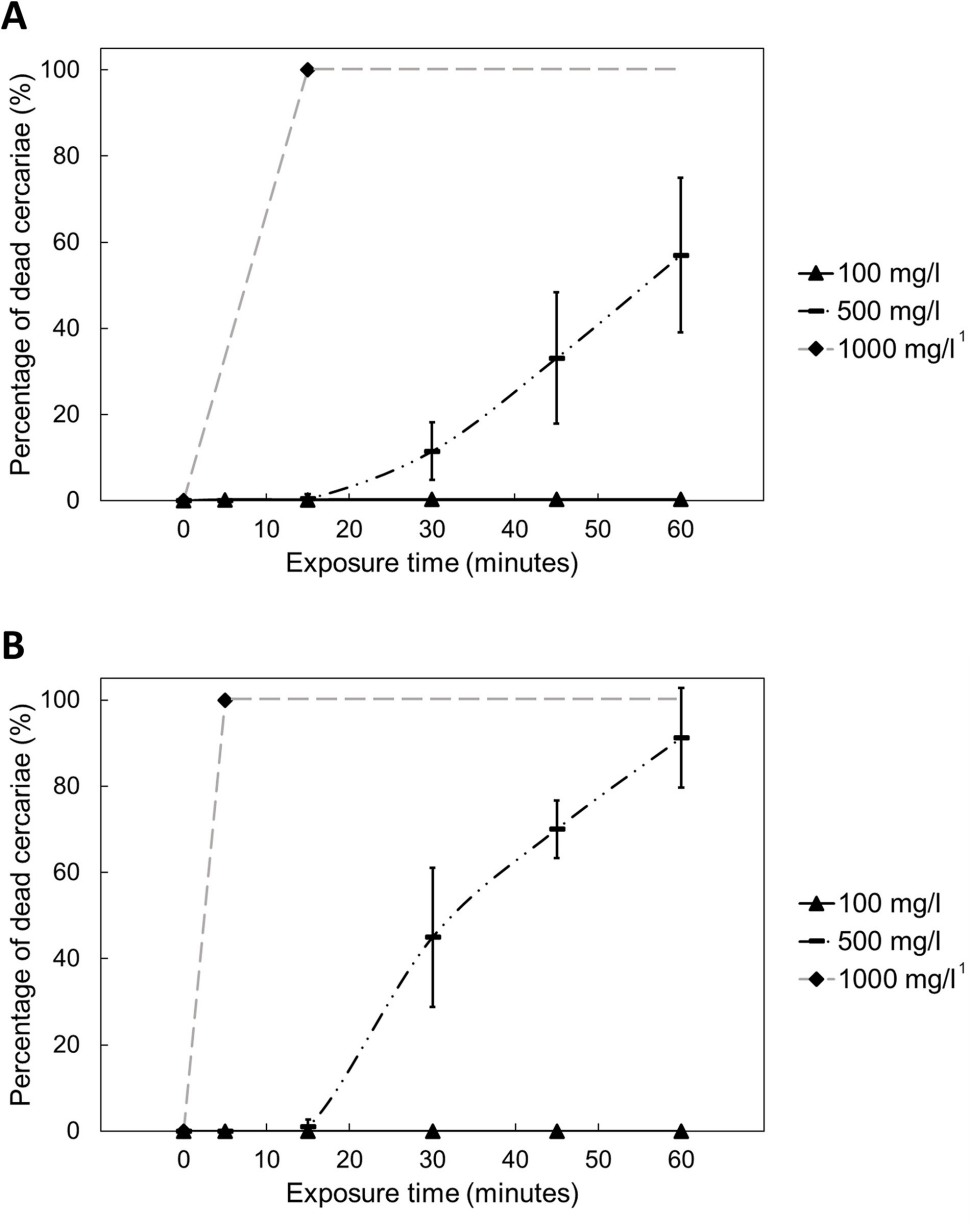
The percentage of dead *S*. *mansoni* cercariae at different concentrations and exposure times of two bar soaps: (A) B29 and (B) Rungu. Each data point represents the average of four replicates with the error bar indicating ±1 standard deviation. ^1^ The long dash grey line was used to connect data points of 0 and 5 minutes or 15 minutes at 1000 mg/L and was extended to 60 minutes, as all cercariae were dead at 5 or 15 minutes of exposure.

The results of the two bar soaps, B29 and Rungu are shown in Fig 2. At 1000 mg/L of Rungu, cercariae gradually sank to the bottom of the beaker and started twitching before eventually dying within 5 minutes. However, 100% cercaria mortality was only achieved in samples of 1000 mg/L B29 at 15 minutes post-exposure, implying the lower lethality of B29 at this concentration. At 100 mg/L, the lowest concentration tested, almost all cercariae survived at 60 minutes post-exposure; however, it was clearly observed that some cercariae started to sink to the beaker bottom and twitch during 5–15 minutes of exposure and then lost the ability to swim upward actively to the surface, which indicates that they were damaged though still alive.

At the middle concentration of 500 mg/L, cercariae began to die substantially after 15 minutes of exposure and eventually reached 57% and 91% mortality at 60 minutes post-exposure to B29 and Rungu, respectively. Similar to powder soap, exposure duration is an important factor of soap lethality since there was a higher proportion of dead cercariae at longer exposure times for both bar soaps. This is also demonstrated in the minimal increases of cercaria mortality observed before 15-minute exposure of 500 mg/l bar soaps, where the average percentages of cercariae alive were 99.5% for B29 and 98.9% for Rungu.

### Comparison of lethality

For the two powder soaps, there was no significant difference in cercaria mortality across all combinations of soap concentrations and exposure times (the statistical results of each Mann-Whitney *U* test can be found in S3 File). This suggests that the two powder soaps tested have similar lethality.

It seems that the anti-bacterial soap, Rungu, was more lethal than B29 since the percentages of dead cercariae were significantly higher than that of B29 at 30 minutes and 45 minutes post-exposure of 500 mg/L (at 30 minutes, Mann-Whitney *U* test, *p* = 0.029; at 45 minutes, Mann-Whitney *U* test, *p* = 0.029), though there was no significant difference at 15 minutes and 60 minutes (at 15 minutes, Mann-Whitney *U* test, *p* = 0.714; at 60 minutes, Mann-Whitney *U* test, *p* = 0.057).

In general, powder soap was more lethal than bar soap. At 100 mg/L, > 99.7% of cercariae survived at 60 minutes post-exposure to two bar soaps while all cercariae died at the same exposure condition of powder soaps. However, this difference was smaller at the highest concentration 1000 mg/L since both powder soaps and the bar soap Rungu killed all cercariae within 5 minutes. Also, when comparing 75 mg/L powder soaps with 500 mg/L bar soaps, these two concentrations demonstrated non-significant lethal effects on cercariae at 60 minutes (Mann-Whitney *U* test, *p* = 0.442), implying that a lower concentration of powder soap was required to reach the similar lethality of bar soap.

## Discussion

The results demonstrate that all four soaps are able to kill *S*. *mansoni* cercariae, and this lethal effect of soap on cercariae is strongly related to soap concentration and exposure time. For the same soap, a longer time is needed to kill the same percentage of cercariae when soap concentration is lower, which confirms the finding of a previous systematic review [21].

Our results also show that powder soap has higher lethality towards cercariae compared to bar soap, which was also reported in a previous study which compared five commercial powder soaps with one local bar soap in Nigeria [25]. Previous research on the anti-bacterial activity also found that powder detergent (Surf excel) was more effective at inhibiting the growth of five bacterial strains than bar soap (Lifebuoy Green), which might be because some chemicals in the powder detergent can improve the anti-bacterial effect [39]. Therefore, the difference in the lethality of soap ingredients may be responsible for the different lethal effects of the two soap types.

The active anti-bacterial component of Rungu is irgasan DP 300, also known as triclosan, which has been widely used in hygiene products such as hand soaps, shower gels, dishwashing soaps and toothpastes [33,40]. One study has shown that repeated handwashing with the bar soaps containing this chemical demonstrated a significant protection against natural skin bacteria in comparison with unmedicated bar soap [41]. Our results show that this anti-bacterial bar soap Rungu was only statistically more lethal than the other bar soap at 30 minutes and 45 minutes post-exposure to 500 mg/L soap, suggesting there is likely limited extra protection provided by anti-bacterial soap.

Two previous studies investigated the lethality of the powder soap Omo, both of which reported an exposure time of 2 minutes to kill all cercariae at 100 ppm [24,25]. However, our results demonstrate that it took 60 minutes to reach 100% cercarial mortality at this concentration, 30 times longer than previously reported. The previous studies also found that a concentration of 10 mg/L was able to kill all cercariae after approximately 60 minutes, whereas we found that almost all cercariae swam actively at 60 minutes post-exposure at this concentration. Due to the unclear protocols of determining cercarial death in those two studies, a less strict protocol might have been used, leading to the higher lethality of Omo in their results.

A few other studies have also determined the adverse effect of local soaps including endod berries and one local soap in Nigeria [25–28]. Endod (*Phytolacca dodecandra*) berries are a traditional local soap widely used in Ethiopia that can be used to wash clothes [42]. One study demonstrated that all *S*. *haematobium* cercariae died at 100 ppm after 60 minutes of exposure, which is similar to our powder soap results [27].

The water used in the experiments is Volvic bottled water. This bottled water contains mineral components such as calcium, magnesium, sodium and potassium and has the pH of 7 [38] which fall into the ranges of those water parameters in African water bodies [34–37]. However, it should be noted that water chemistry may also influence soap lethality. For example, one study has found that the lethal effect of soap on *S*. *mansoni* cercariae increased with the increase in water hardness [24], though it is unclear how the researchers controlled the level of water hardness. Considering the wide ranges of water parameters reported in natural water bodies, Volvic was selected and used as a convenient standard in our research. Increasing water temperature may affect the lethality of soap by accelerating the chemical reaction between soap and cercariae, similar to the chlorine disinfection of cercariae [8], and by enhancing the solubility of soap in water [43]. Future studies can investigate the soap lethality under different water parameters and use natural water samples to compare with the results found in the laboratory.

Soap inactivates microbes by dissolving their lipid membranes and causing the release of their intracellular contents [44]. Schistosome cercariae are covered by a single unit membrane which is made of lipid and an immunogenic glycocalyx which may contain lipid [45]. Soap molecules normally have one hydrophilic head which bonds with water and a hydrophobic tail which bonds with oil and fat. It is possible that soap kills schistosome cercariae in the same way as inactivating other microbes, i.e. it is possible that those molecules destroy cercariae by bonding with the lipid membrane and glycocalyx of cercariae and then releasing the intracellular contents. The ruptured membrane and glycocalyx of cercariae when exposed to other chemicals such as chlorine and hinokitiol [46,47] were reported, though this possible damage caused by soap was not observed under a microscope.

As discussed in a review on the efficacy of soap against cercariae, field studies on interventions with soap use demonstrated conflicting results, which suggests the importance of laboratory results obtained from well-controlled experiments [21]. Based on the lethal effect explored in this experimental study, all soaps killed cercariae within 15 minutes, suggesting the potential impact of improving soap use as a long-term complementary intervention. However, soap is unlikely to provide full protection even if it is used in all water-contact activities.

Unilever recommends dissolving one cup of Omo hand washing powder (one cup ≈ 114 g) in 20 L bucket of water [48], producing a powder soap concentration of 5700 mg/L. Similarly, The Centre for Disease Control and Prevention (CDC) recommends 5000 mg/L when preparing soapy water for cleaning surfaces or objects [49]. Since all cercariae died after 5-minute exposure of 1000 mg/L powder soap in our experiments, the powder soap solution should be cercaria-free and safe to use at 5 minutes after preparation.

As previously mentioned in the methodology, the average bar soap concentration per hand wash was estimated to be approximately 1000 mg/L (details in S1 File). The highest soap concentration is achieved the moment soap is applied on hands and rubbed before rinsing, and the concentration gradually decreases during rinsing before finally reaching zero in the end. Therefore, the actual soap concentration during handwashing can be either much higher than or much lower than the average soap concentration estimated here, leading to the varied protection at each time point during exposure. The varied protection during exposure applies to other water-contact activities including laundry, bathing and dishwashing as all these activities involve the rinsing process which lowers the soap concentration. Since soap at a high concentration of 1000 mg/L is not able to kill cercariae instantly and soap concentration changes with rinsing, soap is not likely to provide sufficient protection under the mechanism of killing cercariae. Nevertheless, it is possible that even low concentrations of soap could render cercariae unable to penetrate skin.

The species tested is *S*. *mansoni*, one of the three main species of schistosomes that infect humans [29,30]. However, it remains unknown whether there is species variability in soap, and therefore *S*. *haematobium* and *S*. *japonicum* should be considered in future studies. Apart from the four soaps tested in this study, there are also other soaps used by local people living in the endemic village. Future research can investigate more soaps especially other soap types such as local soap (e.g. Endod berries in Ethiopia) or liquid soap in endemic regions to study the lethality of different soaps that people use. Additionally, all the experiments in this study were carried out in the laboratory, and it remains unclear whether these laboratory results will align with the field results. Therefore, field research is needed using natural water samples and cercariae released from “wild” snails in disease-endemic areas. If soap use is promoted to prevent schistosomiasis infection in the future, it is important to conduct studies on evaluating the environmental impact of soap at the concentrations proposed and the availability and affordability of soap in endemic regions.

It is worth noting that the adverse endpoint of cercariae assessed in this study is mortality which was determined by cercaria motility and morphology. Our results showed that some cercariae were clearly damaged when exposed to 100 mg/L bar soap since they started to fall down to the bottom of the beaker since 5 minutes of exposure, twitch and lose the ability to swim back to the surface water, though they were still alive. Therefore, it is important to test whether these damaged but alive cercariae still pose a risk to people during water contact. As summarised in our systematic review, there are two groups of soap protective mechanisms: (1) the adverse effect of soap on different endpoints of cercariae such as mortality and infectivity, (2) soap protects the skin by preventing cercariae from penetrating the skin, developing into adult worms and producing eggs [21]. From a public health standpoint, soap does not necessarily kill cercariae and may still be protective if any potential mechanism above is effective. For example, it is possible that soap protects individuals at lower concentrations and shorter exposure times by making cercariae lose the ability to penetrate skin and develop into adult worms. One study has found that there was a reduction of about 85% worm burden when mouse tails were exposed to cercariae treated with a low concentration of 10 ppm powder soap Omo for 30 minutes [25]. To further explore the possible protection of soap, future studies should investigate other potential protective mechanisms of soap. However, based on measurement of the direct lethality of soap against cercariae alone, this study demonstrated that soap is unlikely to be fully protective under normal usage conditions.

## Conclusions

Our study has determined the quantitative lethal effect of four soaps on *S*. *mansoni* cercariae. The results suggest that these soaps can kill cercariae, and this effect was related to soap concentration and exposure time. Among the two soap types tested in this study, powder soap was more lethal than bar soap, which might be due to the difference in soap ingredients.

Even at a very high concentration of 1000 mg/L, soap did not kill all cercariae instantly. Since the soap concentration during washing activities is highly dynamic, it is likely that soap protection is variable and incomplete. As the adverse endpoint of cercariae determined in this study was death, our results represent the minimum soap protection that people can obtain at each controlled exposure concentration and time. Therefore, it is necessary to investigate whether cercariae are still able to penetrate skin when exposed to low concentrations of soap or soap can protect skin against the penetration of cercariae, which may provide further confidence in the use of soap as a protective measure.

## Supporting information

S1 File Estimation of the average soap concentration during handwashing. (DOCX)

S2 File Photos of dead and living cercariae. (DOCX)

S3 File Table of the statistical results of comparing two powder soaps using the Mann-Whitney *U* test. (DOCX)

S1 Data Percentages of dead cercariae under different experimental conditions. (XLSX)

## References

[1] World Health Organization. Ending the neglect to attain the Sustainable Development Goals: a road map for neglected tropical diseases 2021–2030. Geneva; 2020.

[2] Global Burden of Disease Study 2019. Data Resources. Institute for Health Metrics and Evaluation; 2019. Available: https://ghdx.healthdata.org/gbd-2019

[3] World Health Organization. WHO guideline on control and elimination of human schistosomiasis. Geneva; 2022. 35235279

[4] Freeman MC, Ogden S, Jacobson J, Abbott D, Addiss DG, Amnie AG, et al. Integration of water, sanitation, and hygiene for the prevention and control of neglected tropical diseases: a rationale for inter-sectoral collaboration. PLoS Negl Trop Dis. 2013;7: e2439. doi: 10.1371/journal.pntd.0002439 24086781 PMC3784463

[5] Campbell SJ, Biritwum N-K, Woods G, Velleman Y, Fleming F, Stothard JR. Tailoring Water, Sanitation, and Hygiene (WASH) Targets for Soil-Transmitted Helminthiasis and Schistosomiasis Control. Trends Parasitol. 2018;34: 53–63. doi: 10.1016/j.pt.2017.09.004 29055522

[6] Braun L, Grimes JET, Templeton MR. The effectiveness of water treatment processes against schistosome cercariae: A systematic review. PLoS Negl Trop Dis. 2018;12: e0006364. doi: 10.1371/journal.pntd.0006364 29608589 PMC5903662

[7] Hazell L, Allan F, Emery AM, Templeton MR. Ultraviolet disinfection of Schistosoma mansoni cercariae in water. PLoS Negl Trop Dis. 2021;15: e0009572. doi: 10.1371/journal.pntd.0009572 34228750 PMC8284627

[8] Braun L, Sylivester YD, Zerefa MD, Maru M, Allan F, Zewge F, et al. Chlorination of Schistosoma mansoni cercariae. PLoS Negl Trop Dis. 2020;14: e0008665. doi: 10.1371/journal.pntd.0008665 32822356 PMC7467251

[9] Braun L, Sylivester YD, Zerefa MD, Maru M, Allan F, Zewge F, et al. Parameters for effective sand filtration of Schistosoma mansoni cercariae from water. Water Supply. 2022;22: 1943–1950. doi: 10.2166/ws.2021.312

[10] Hazell L, Braun L, Templeton MR. Ultraviolet sensitivity of WASH (water, sanitation, and hygiene) -related helminths: A systematic review. PLoS Negl Trop Dis. 2019;13: e0007777. doi: 10.1371/journal.pntd.0007777 31536504 PMC6772140

[11] Fulford AJC, Ouma JH, Kariuki HC, Thiongo FW, Klumpp R, Kloos H, et al. Water contact observations in Kenyan communities endemic for schistosomiasis: methodology and patterns of behaviour. Parasitology. 1996;113: 223–241. doi: 10.1017/s0031182000082007 8811848

[12] Reitzug F, Ledien J, Chami GF. Associations of water contact frequency, duration, and activities with schistosome infection risk: A systematic review and meta-analysis. PLoS Negl Trop Dis. 2023;17: e0011377. doi: 10.1371/journal.pntd.0011377 37315020 PMC10266691

[13] Ayabina DV, Clark J, Bayley H, Lamberton PHL, Toor J, Hollingsworth TD. Gender-related differences in prevalence, intensity and associated risk factors of Schistosoma infections in Africa: A systematic review and meta-analysis. PLoS Negl Trop Dis. 2021;15: e0009083. doi: 10.1371/journal.pntd.0009083 34788280 PMC8635327

[14] Friedman M, Wolf R. Chemistry of soaps and detergents: Various types of commercial products and their ingredients. Clin Dermatol. 1996;14: 7–13. doi: 10.1016/0738-081x(95)00102-l 8901393

[15] Coiffard L, Couteau C. Soap and syndets: differences and analogies, sources of great confusion. Eur Rev Med Pharmacol Sci. 2020;24: 11432–11439. doi: 10.26355/eurrev_202011_23637 33215466

[16] Bailey RA, Clark HM, Ferris JP, Krause S, Strong RL. Soaps, synthetic surfactants, and polymers. Chemistry of the Environment. Elsevier; 2002. pp. 193–221. doi: 10.1016/B978-012073461-0/50054-5

[17] Stocks ME, Ogden S, Haddad D, Addiss DG, McGuire C, Freeman MC. Effect of Water, Sanitation, and Hygiene on the Prevention of Trachoma: A Systematic Review and Meta-Analysis. PLoS Med. 2014;11: e1001605. doi: 10.1371/journal.pmed.1001605 24586120 PMC3934994

[18] Curtis V, Cairncross S. Effect of washing hands with soap on diarrhoea risk in the community: a systematic review. Lancet Infect Dis. 2003;3: 275–281. doi: 10.1016/s1473-3099(03)00606-6 12726975

[19] Aiello AE, Coulborn RM, Perez V, Larson EL. Effect of Hand Hygiene on Infectious Disease Risk in the Community Setting: A Meta-Analysis. Am J Public Health. 2008;98: 1372–1381. doi: 10.2105/AJPH.2007.124610 18556606 PMC2446461

[20] Howard G, Blogh C, Goldstein G, Morgan J, Prüss-Üstün A, Shaw R, et al. Healthy villages: a guide for communities and community health workers. Geneva: World Health Organization; 2002. Available: https://apps.who.int/iris/handle/10665/42456

[21] Zhang J, Pitol AK, Braun L, Hazell L, Templeton MR. The efficacy of soap against schistosome cercariae: A systematic review. PLoS Negl Trop Dis. 2022;16: e0010820. doi: 10.1371/journal.pntd.0010820 36191022 PMC9560551

[22] Edungbola LD. The protective potentials of 4-chloro-3, 5-xylenol (Dettol) against Mansonian schistosomiasis. Afr J Med Med Sci. 1980;9: 97–102. Available: http://www.ncbi.nlm.nih.gov/pubmed/6283866 6283866

[23] Pacheco G, Jansen J. [Destruction of Schistosoma mansoni cercariae with soaps combined with dyes]. Bras Med. 1951;65: 301–4, includes English transl. Available: http://www.ncbi.nlm.nih.gov/pubmed/14935011 14935011

[24] Mimpfoundi R, Dupouy J. [Action of various detergents in use in Cameroon on the vitality of Schistosoma mansoni cercariae: influence of the hardness factor of water]. C R Seances Soc Biol Fil. 1983;177: 338–346. Available: http://www.ncbi.nlm.nih.gov/pubmed/6225490 6225490

[25] Okwuosa VN, Osuala FO. Toxicity of washing soaps to Schistosoma mansoni cercariae and effects of sublethal concentrations on infectivity in mice. Appl Parasitol. 1993;34: 69–75. Available: http://www.ncbi.nlm.nih.gov/pubmed/8508221 8508221

[26] Birrie H, Balcha F, Erko B, Bezuneh A, Gemeda N. Investigation into the cercariacidal and miracidiacidal properties of Endod (Phytolacca dodecandra) berries (type 44). East Afr Med J. 1998;75: 311–4. Available: http://www.ncbi.nlm.nih.gov/pubmed/9747006 9747006

[27] Lemma A. Laboratory and field evaluation of the molluscicidal properties of Phytolacca dodecandra. Bull World Health Organ. 1970;42: 597–612. Available: http://www.ncbi.nlm.nih.gov/pubmed/5310955 5310955 PMC2427471

[28] Monkiedje A, Anderson AC, Englande AJ. Acute toxicity of Phytolacca dodecandra (Endod-S) and Niclosamide to snails, Schistosoma mansoni cercaria, Tilapia fish, and soil microorganisms. Environ Toxicol Water Qual. 1991;6: 405–413. doi: 10.1002/tox.2530060405

[29] Gryseels B, Polman K, Clerinx J, Kestens L. Human schistosomiasis. Lancet. 2006;368: 1106–18. doi: 10.1016/S0140-6736(06)69440-3 16997665

[30] McManus DP, Dunne DW, Sacko M, Utzinger J, Vennervald BJ, Zhou X-N. Schistosomiasis. Nat Rev Dis Primers. 2018;4: 13. doi: 10.1038/s41572-018-0013-8 30093684

[31] Ulmer MJ. Notes on rearing of snails in the laboratory. In: MacInnis AJ, Voge M, editors. Experiments and techniques in parasitology. W. H. Freeman and Company (San Francisco); 1970. pp. 143–144.

[32] Braun L, Hazell L, Webb AJ, Allan F, Emery AM, Templeton MR. Determining the viability of Schistosoma mansoni cercariae using fluorescence assays: An application for water treatment. PLoS Negl Trop Dis. 2020;14: e0008176. doi: 10.1371/journal.pntd.0008176 32214320 PMC7138324

[33] Campbell L, Zirwas MJ. Triclosan. Dermatitis. 2006;17: 204–7. Available: http://www.ncbi.nlm.nih.gov/pubmed/17150172 17150172

[34] Nakirya D, James OO, Fredrick JM. Microbial safety assessment of recreation water at Lake Nabugabo, Uganda. Afr J Environ Sci Tech. 2015;9: 773–782. doi: 10.5897/AJEST2015.1979

[35] Karume K, Bagalwa M, Bagula E, Yalire M, Habakaramo P, Byamukama J, et al. Water Quality in and around Lake Edward Basin of the Greater Virunga Landscape, D. R. Congo Side. J Environ Prot (Irvine, Calif). 2019;10: 1174–1193. doi: 10.4236/jep.2019.109070

[36] Athuman CB. The Multivariate Statistical Analysis of the Environmental Pollutants at Lake Nyamagoma. Journal of Natural Sciences Research. 2013;3: 96–103.

[37] Nimusiima D, Byamugisha D, Omara T, Ntambi E. Physicochemical and Microbial Quality of Water from the Ugandan Stretch of the Kagera Transboundary River. Limnological Review. 2023;23: 157–176. doi: 10.3390/limnolrev23030010

[38] Volvic UK: Natural Mineral Water 1.5L. [cited 14 Jan 2024]. Available: https://www.volvic.co.uk/products/natural-mineral-water/volvic-natural-mineral-water/natural-mineral-water-15l

[39] Bhat R, Prajna, Menezez VP, Shetty PR. Antimicrobial Activities of Soap and Detergents. 2012. Available: www.soeagra.com/abr.htm

[40] Jones RD, Jampani HB, Newman JL, Lee AS. Triclosan: A review of effectiveness and safety in health care settings. Am J Infect Control. 2000;28: 184–196. doi: 10.1067/mic.2000.102378 10760227

[41] Lilly HA, Lowbury EJ. Disinfection of the skin with detergent preparations of Irgasan DP 300 and other antiseptics. Br Med J. 1974;4: 372–4. doi: 10.1136/bmj.4.5941.372 4609556 PMC1612462

[42] Esser KB, Semagn K, Wolde-Yohannes L. Medicinal use and social status of the soap berry endod (Phytolacca dodecandra) in Ethiopia. J Ethnopharmacol. 2003;85: 269–77. doi: 10.1016/s0378-8741(03)00007-2 12639751

[43] McBain JW, Sierichs WC. The solubility of sodium and potassium soaps and the phase diagrams of aqueous potassium soaps. J Am Oil Chem Soc. 1948;25: 221–225. doi: 10.1007/BF02645899

[44] Jing JLJ, Pei Yi T, Bose RJC, McCarthy JR, Tharmalingam N, Madheswaran T. Hand Sanitizers: A Review on Formulation Aspects, Adverse Effects, and Regulations. Int J Environ Res Public Health. 2020;17: 3326. doi: 10.3390/ijerph17093326 32403261 PMC7246736

[45] Samuelson JC, Caulfield JP. The cercarial glycocalyx of Schistosoma mansoni. J Cell Biol. 1985;100: 1423–1434. doi: 10.1083/jcb.100.5.1423 2985622 PMC2113874

[46] Braun L. The effectiveness of water treatment processes against schistosome cercariae. PhD thesis. Imperial College London. 2021. 10.1371/journal.pntd.0006364 PMC5903662 29608589

[47] Chisty MM, Nargis M, Inaba T, Ishita K, Osanai A, Kamiya H. Transmission electron microscopy of Schistosoma mansoni cercariae treated with hinokitiol (beta-thujaplicin), a compound for potential skin application against cercarial penetration. Tohoku J Exp Med. 2004;202: 63–7. doi: 10.1620/tjem.202.63 14738326

[48] Unilever. Omo Hand Washing Powder 9 kg. In: 2024 [Internet]. [cited 27 Jan 2024]. Available: https://www.unileverprofessional.co.za/products/4/2/omo-hand-washing-powder

[49] Centre for Disease Control and Prevention. How to Make Soapy Water Cleaning Solution. [cited 22 Apr 2024]. Available: https://www.cdc.gov/global-water-sanitation-hygiene/media/pdfs/334204-E_NCEZID_IG_CleaningSolution-p.pdf

