# Supplementary material for: The lethal effect of soap on Schistosoma mansoni cercariae in water
Source: PLoS Negl Trop Dis. 2024 Jul 29;18(7):e0012372. doi: 10.1371/journal.pntd.0012372 (PMC11309484; doi:10.1371/journal.pntd.0012372)
Supplement: S1 File — (DOCX) [file pntd.0012372.s001.docx]

**Article title:** The lethal effect of soap on *Schistosoma mansoni* cercariae in water

**Authors:** Jiaodi Zhang, Ana K. Pitol, Safari Kinung’hi, Teckla Angelo, Aidan M. Emery, Adam Cieplinski, Michael R. Templeton, Laura Braun

**S1 File. Estimation of the average soap concentration during handwashing.**

**Methods**

A laboratory experiment was carried out to estimate the average soap concentration during washing hands. The methods are as follows:

A common commercial bar soap was weighed first to obtain the soap mass. The handwashing process followed the guidelines proposed by Centers for Disease Control and Prevention [1]. Hands were wetted with running tap water first with the tap closed afterwards, and applied with the soap. Then, hands were rubbed with soap to form lather and scrubbed for at least 20 seconds. With tap on, hands were rinsed under running water with a basin below to collect water during rinsing hands. The water in the basin was transferred to a measuring cylinder to read the volume of water. The bar soap was weighed again 24 hours later to obtain the mass after handwashing. The average soap concentration was calculated as below:

$$Average soap concentration per hand wash= \frac{Soap weight before handwashing-Soap weight after handwashing}{The volume of rinsing water}$$

This experiment was carried out by three people and each person repeated three times.

**Results**

The average weight of soap used per hand wash was 0.27±0.09 g and the average volume of water consumed was 258±99 mL. It was calculated that the average soap concentration during handwashing was 1075±223 mg/L.

Reference:

1. Centre for Disease Control and Prevention (CDC). When and How to Wash Your Hands. 2024 [cited 22 Jan 2024]. Available: https://www.cdc.gov/handwashing/when-how-handwashing.html
