# Supplementary material for: The lethal effect of soap on Schistosoma mansoni cercariae in water
Source: PLoS Negl Trop Dis. 2024 Jul 29;18(7):e0012372. doi: 10.1371/journal.pntd.0012372 (PMC11309484; doi:10.1371/journal.pntd.0012372)

**Article title:** The lethal effect of soap on *Schistosoma mansoni* cercariae in water

**Authors:** Jiaodi Zhang, Ana K. Pitol, Safari Kinung’hi, Teckla Angelo, Aidan M. Emery, Adam Cieplinski, Michael R. Templeton, Laura Braun

**S2 File. Photos of dead and living cercariae.**

The following characteristics of cercariae were examined to determine whether a cercaria is dead or not:

1. a cessation of motility
2. everted suckers
3. a fully relaxed tail
4. a slightly sharp-edge of the head-tail-junction on the head side

Examples of a dead cercarial (A) head and (B) tail in the soap sample:


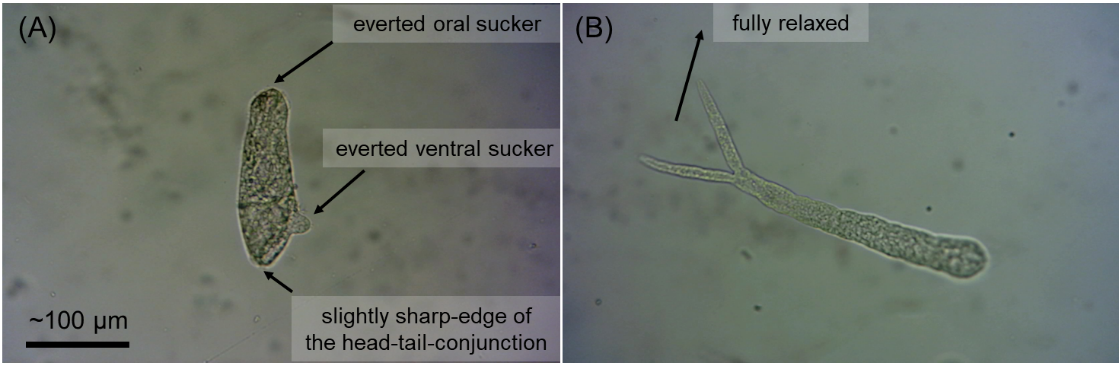


Examples of a few living cercarae in the control samples:


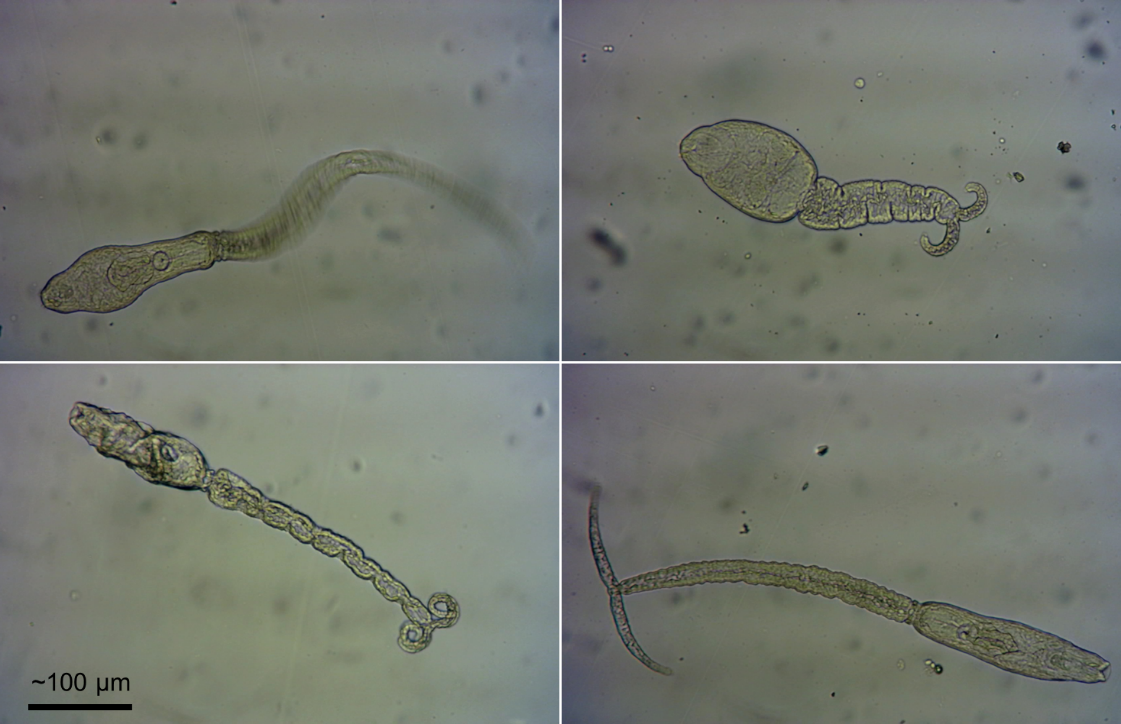

Supplement: S2 File — (DOCX) [file pntd.0012372.s002.docx]
