# Supplementary material for: The lethal effect of soap on Schistosoma mansoni cercariae in water
Source: PLoS Negl Trop Dis. 2024 Jul 29;18(7):e0012372. doi: 10.1371/journal.pntd.0012372 (PMC11309484; doi:10.1371/journal.pntd.0012372)
Supplement: S3 File — (DOCX) [file pntd.0012372.s003.docx]

**Article title:** The lethal effect of soap on *Schistosoma mansoni* cercariae in water

**Authors:** Jiaodi Zhang, Ana K. Pitol, Safari Kinung’hi, Teckla Angelo, Aidan M. Emery, Adam Cieplinski, Michael R. Templeton, Laura Braun

**S3 File. Table of the statistical results of comparing two powder soaps using the Mann-Whitney *U* test.**

There are four percentages of dead cercariae (%) which are obtained from the four independent replicates at an experimental condition (i.e. a soap concentration and an exposure time), and these four percentages at an experiment condition were compared with those percentages at another experimental condition using the Mann-Whitney *U* test. The results of *p* values obtained from each Mann-Whitney *U* test are summarised in the table below.

| Soap concentration  Exposure time | 10 mg/L | 50 mg/L | 75 mg/L | 100 mg/L | 1000 mg/L |
| --- | --- | --- | --- | --- | --- |
| 0 minutes | 1.000 | 1.000 | 1.000 | 1.000 | 1.000 |
| 5 minutes | 0.429 | 1.000 | 0.429 | 0.200 | 1.000 |
| 15 minutes | 0.429 | 0.057 | 0.114 | 0.343 | N/A |
| 30 minutes | 0.429 | 0.686 | 0.114 | 0.343 | N/A |
| 45 minutes | 1.000 | 0.686 | 0.486 | 0.486 | N/A |
| 60 minutes | 0.486 | 0.886 | 0.343 | 1.000 | N/A |

* Exact Significance (2-tailed) values were reported as *p* values.
